# Colon Capsule Endoscopy in the Diagnosis of Colon Polyps: Who Needs a Colonoscopy?

**DOI:** 10.3390/diagnostics12092093

**Published:** 2022-08-29

**Authors:** Apostolos Koffas, Apostolis Papaefthymiou, Faidon-Marios Laskaratos, Andreas Kapsoritakis, Owen Epstein

**Affiliations:** 1 Department of Gastroenterology, University Hospital of Larissa, Mezourlo, 41110 Larissa, Greece; 2 Wolfson Unit for Endoscopy, St Mark’s Hospital, London HA1 3UJ, UK; 3 Institute for Minimally Invasive Gastroenterology, Royal Free London, London NW3 2QG, UK

**Keywords:** colon capsule endoscopy, colon polyps, colorectal cancer screening

## Abstract

Colon screening programs have reduced colon cancer mortality. Population screening should be minimally invasive, safe, acceptably sensitive, cost-effective, and scalable. The range of screening modalities include guaiac or immunochemical fecal occult blood testing and CT colonography and colonoscopy. A number of carefully controlled studies concur that second-generation capsule endoscopy has excellent sensitivity for polyp detection and a high negative predictive value. Colon capsules fulfill the screening expectation of safety, high sensitivity for polyp detection, and patient acceptance, and appear to straddle the divide between occult blood testing and colonoscopy. While meeting these criteria, there remains the challenges of scaling, capsule practitioner training, resource allocation, and implementing change of practice. Like CT colonography, capsule screening presents the clinician with a decision on the threshold for colonoscopy referral. Overall, colon capsules are an invaluable tool in polyp detection and colon screening and offer a filter that determines “who needs a colonoscopy?”.

## 1. Introduction

Colorectal cancer (CRC) represents a leading cause of cancer-related death in men and women globally, causing 600,000 deaths each year [1]. Almost all CRC cases arise from mutations occurring in benign colonic polyps, and prognosis is largely dependent on tumor stage at the time of diagnosis. Since progression from normal mucosa to invasive cancer can take 10 to 15 years, early diagnosis and removal of precancerous polyps is highly effective in preventing the development of CRC. CRC screening for polyps in average-risk adults has been universally accepted. Screening modalities include testing for fecal occult blood testing (FOBT), fecal immunochemical testing (FIT), flexible sigmoidoscopy, virtual colonoscopy, and traditional colonoscopy. Implementation of screening programs has contributed to a reduction in CRC mortality, although there is no effect on all-cause mortality [2].

Population screening should be minimally invasive, safe, acceptably sensitive, cost-effective, and scalable. In some healthcare settings, colonoscopy is the preferred screening modality. The procedure is the recognized gold standard for the diagnosis of colon polyps and early-stage cancer but fails the criteria for acceptable and widespread population screening [3]. The procedure requires intensive bowel preparation, day-case admission, intravenous analgesia, and sedation or light anesthesia, and there is also a small risk of severe adverse events, including bowel perforation, hemorrhage, and cardiovascular events. Examinations may be incomplete and even experienced colonoscopists reporting back-to-back studies recognize polyp miss rates exceeding 20%, especially for smaller (<5 mm) polyps [4,5].

In 2000, Gavriel Iddan and colleagues published the first use of a wireless capsule endoscope designed to illuminate and transmit images of the small intestine. Microelectronics freed the device from external cabling and fiber-optic bundles, and the capsule was small enough to swallow with a sip of water. The images were transmitted using radiotelemetry and stored on an external recording device. Successive generations of hardware and software innovation followed, resulting in progressive improvements in image resolution, power consumption, and ease of use [6].

Minimally-invasive colon capsule endoscopy (CCE) offers a tantalizing option for patients undergoing screening, surveillance, and investigation of lower gastrointestinal symptoms. Straddling fecal occult blood and FIT testing and colonoscopy, the investigation is painless, easily administered, allows direct visualization of the colonic mucosa, and is almost free of serious procedure related adverse events. However, the potential of CCE screening presents a number of challenges, including extending battery life to ensure study completion rates equivalent to those expected of colonoscopy, the requirement for consistent high-quality colon cleansing, and the need to rapidly train large numbers of capsule readers.

This review summarizes key CCE publications and discusses the possible role of this new colon imaging device in CRC screening.

## 2. 1st-Generation Colon Capsule Endoscopy and Polyp Detection

In 2006, the first-generation of colon capsule CCE-1 [Given Imaging (Yoqneam, Israel)] was introduced [7,8]. This capsule differed from the small bowel capsule, requiring a slightly larger outer casing (31 mm × 11 mm), housing a camera and light source at each end that was capable of surveying a front and rear visual field of 156°. The camera acquired mucosal images at a rate of four frames per second and battery life was extended to 10–11 h. Following the introduction of CCE-1, several studies were reported assessing the sensitivity and specificity of CCE in detecting colonic polypoid and non-polypoid lesions [7,8,9,10,11,12,13,14,15,16,17,18].

Rokkas et al. reported a meta-analysis of polyp detection with CCE-1 in detecting colonic polyps. The pooled data showed per-patient CCE sensitivity of 73% (95% CI, 68–77%) and specificity of 89% (95% CI, 81–94%). For polyps >6 mm in size or 3 or more polyps of any size, the respective values were 69% (95% CI, 62–75%) and 86% (95% CI, 82–90%) [19]. Spada et al. also reported a systematic review and meta-analysis of the accuracy of CCE-1 to detect colorectal polyps. The report included eight studies comprising 837 patients. The prevalence of polyps was 57%. The CCE-1 sensitivity and specificity for reporting polyps of any size was 71% and 75%, respectively [20].

## 3. 2nd-Generation Colon Capsule Endoscopy and Polyp Detection

**Figure 1:**
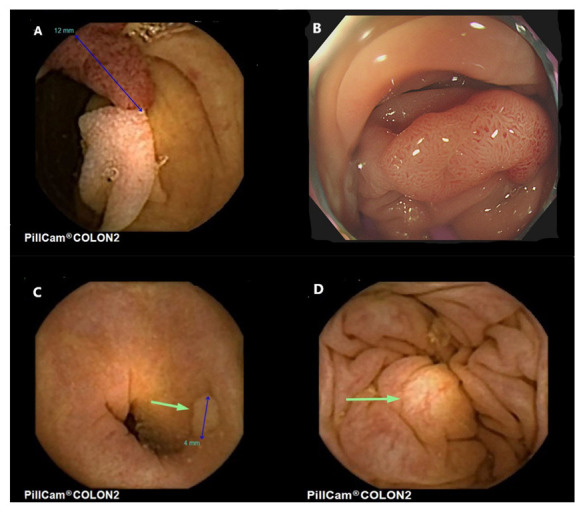
Colon polyps seen from colon capsule endoscopy. (**A**). Pedunculated adenomatous polyp in the sigmoid colon, (**B**). The same pedunculated polyp as seen at colonoscopy. Histology revealed an adenoma with low grade dysplasia, (**C**). Diminutive hyperplastic polyp, (**D**). A submucosal lesion in the colon with yellowish appearance consistent with a probable lipoma.

**Figure 2:**
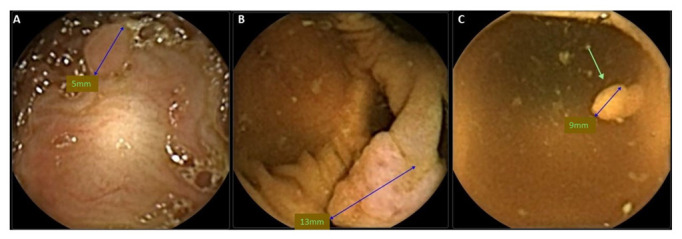
Colon polyps seen from colon capsule endoscopy. (**A**). Diminutive hyperplastic polyp, (**B**). 13-mm sessile polyp, and (**C**). small, pedunculated adenomatous polyp.

When compared to colonoscopy, this first-generation capsule revealed poor sensitivity and specificity. This spurred the development of a second-generation capsule (CCE-2), which was re-designed to address the shortcomings of CCE-1. Launched in 2009, CCE-2 was slightly larger (31.5 mm × 11.6 mm) with the camera at each end being capable of surveying a field of 172°, thereby achieving an overall coverage of almost 360°. The integration of a bidirectional information flow algorithm linking the capsule with the DR3 data recorder allowed modulation of the frame rate at either 4 or 35 frames per second, depending on whether the capsule was stationary or moving. The adaptive frame rate, together with a “sleep” mode in the stomach, resulted in the extension of battery life to around 14 h [21]. The technical characteristics of second-generation colon capsule are summarized in Table 1. Examples of colon polyps detected with CCE are shown in Figure 1 and Figure 2.

In 2009, Eliakim et al. reported the first controlled CCE-2 study, which comprised 98 patients. Each patient underwent a colonoscopy, followed within 10 h by a CCE-2 examination. The per-patient CCE-2 sensitivity for polyps ≥6 mm was 89% [95% CI, 70–97%], and for ≥10 mm it was 88% (95% CI, 56–98%), with specificities of 76% (95% CI, 72–78%) and 89% (95% CI, 86–90%), respectively [22]. In 2011, Spada et al. reported a multicenter prospective European study of 109 patients where CCE-2 was compared with conventional “gold standard” colonoscopy. The per-patient sensitivity of CCE-2 for polyps at least 6 mm in size was 84% (95% CI, 74–95%), and for polyps at least 10 mm in size it was 88% (95% CI, 76–99%), with a specificity of 64% and 95%, respectively [23]. These studies signaled the markedly improved sensitivity and accuracy of CCE-2 compared to CCE-1. Several subsequent controlled studies have reported the efficacy of CCE-2 in identifying colonic polyps [24,25,26,27,28,29,30,31,32,33,34,35,36,37,38,39,40,41,42,43,44,45,46,47,48,49,50,51,52].

In one of the largest studies to date, Rex et al. reported the accuracy of CCE-2 in detecting polyps that are 6 mm or larger in an average-risk screening population. Among 695 patients analyzed, CCE-2 identified subjects with at least one polyp ≥6 mm with 81% sensitivity (95% CI, 77–84%) and 93% specificity (95% CI, 91–95%), and polyps ≥10 mm were identified with 80% sensitivity (95% CI, 74–86%) and 97% specificity (95% CI, 96–98%). Notably, CCE-2 identified individuals with at least one adenoma measuring at least 6 mm or larger with 88% sensitivity (95% CI, 82–93%) and 82% specificity (95% CI, 80–83%), and ones measuring 10 mm or larger with 92% sensitivity (95% CI, 82–97%) and 95% specificity (95% CI, 94–95%) [28].

In 2016, Spada et al. conducted a systematic review and meta-analysis of the accuracy of first- and second-generation CCE in the detection of colorectal polyps. The report included 14 studies comprising data from 1128 individuals assessed with CCE-1 and 1292 with CCE-2. CCE-2 detected polyps ≥6 mm with a sensitivity of 86% (95% CI, 82–89%) and specificity of 88.1% (95% CI, 74.2–95.0%). Considering polyps ≥10 mm, CCE-2 demonstrated an 87% sensitivity (95% CI, 81–91%) and 95.3% specificity (95% CI, 91.5–97.5%). This meta-analysis established clear evidence that unlike CCE-1, CCE-2 achieved acceptably high detection rates [53].

In a more recent 2021 systematic review and meta-analysis, the diagnostic accuracy of CCE was compared with colonoscopy in polyp detection. The mean 95% CI sensitivity, specificity, and diagnostic odds ratio were: 0.85 (0.73–0.92), 0.85 (0.70–0.93), and 30.5 (16.2–57.2), respectively, for polyps of any size; 0.87 (0.82–0.90), 0.95 (0.92–0.97), and 136.0 (70.6–262.1), respectively, for polyps ≥ 10 mm; and 0.87 (0.83–0.90), 0.88 (0.75–0.95), and 51.1 (19.8–131.8), respectively, for polyps ≥ 6 mm. These findings illustrated that CCE had a high sensitivity and specificity for per-patient polyps that was comparable with standard colonoscopy [54].

In another interesting systematic review and meta-analysis of clinical trials, the high diagnostic accuracy of CCE was once again confirmed in adequately-cleaned bowels. The pooled sensitivities and specificities for polyps ≥ 6 mm were 87% (95% CI: 83–90%) and 87% (95% CI: 76–93%) in 8 studies, respectively. For polyps ≥ 10 mm, the pooled estimates for sensitivities and specificities were 87% (95% CI: 83–90%) and 95% (95% CI: 92–97%) in 9 studies, respectively [55]. The recent systematic reviews and meta-analyses are summarized in Table 2.

Lastly, in 2022, Vuik et al. reported the prevalence of gastrointestinal abnormalities in 462 asymptomatic patients that were invited to undergo CCE. In 46 (10.2%) of these asymptomatic individuals, there were significant findings in the colon, i.e., colonic polyps—or CRC in one [56]. This study clearly indicates that whole bowel imaging with CCE reveals that no digestive tract is blemish-free and clinically-significant polyps can be found in a non-negligible number of asymptomatic individuals. 

## 4. CCE-2 and Flat Polyps

There are few reports regarding the accuracy of CCE in the detection of non-polypoid lesions such as flat polyps, lateral spreading tumours, diverticulosis, angiodysplasia, and inflammatory colitis.

Flat polyps are of special interest since these lesions may harbor a higher risk of early malignant transformation [59,60,61,62]. Spada et al. conducted an evaluation of patients enrolled in prospective comparative trials that used the conventional colonoscopy as gold standard. Twenty-seven polyps ≥6 mm were detected in 16 patients by optical colonoscopy. According to the Paris polyp classification, 15 out of 27 (55.5%) were classified as IIA lesions (i.e., non-polypoid-superficial, elevated lesions) and 12 (44.5%) as IS lesions (i.e., polypoid-protruded, sessile lesions). Twenty-five polyps were detected by CCE and 24 (96%) were classified as polypoid lesions with only a single lesion (4%) classified as non-polypoid. The authors commented that all the IIA lesions detected by conventional colonoscopy were classified as polypoid-protruded-sessile lesions by CCE and concluded that the Paris classification does not seem applicable to CCE [63]. It has been suggested that during conventional colonoscopy, gas insufflation distends the bowel wall, thereby flattening the polyps. On CCE, because of the undistended bowel, these same polyps have the appearance of conventional protruding lesions. 

A single center study performed by Igawa et al. attempted to evaluate the sensitivity and specificity of CCE for detecting lateral spreading tumors (LST). The authors performed a prospective study comparing CCE-2 with optical colonoscopy in 21 patients with LSTs diagnosed during a 3-month period prior to capsule endoscopy. The sensitivity and specificity of CCE-2 for detecting LSTs was 81% and 100%, respectively. For detecting LST-granular type and LST-non-granular type, the sensitivity and specificity were 71% and 100% and 86% and 100%, respectively. The authors concluded that CCE-2 was less sensitive in detecting LSTs than optical colonoscopy, especially for LSTs located in the right colon. Nevertheless, the study was limited to a small sample size [32]. 

All these studies assessing the accuracy of CCE in diagnosing colonic polypoid lesions clearly demonstrate that CCE-2 has sufficient sensitivity and specificity for its use to be considered for colon polyp screening and surveillance and determining who needs an interventional colonoscopy.

## 5. The Role of Colon Capsule Endoscopy in Screening for Advanced Colorectal Neoplasia (Advanced Adenomas and CRC)

Colon screening programs designed to detect adenomas and early-stage cancer have been widely adopted and the optimal age to initiate screening has progressively decreased, adding to demand for screening services. Current consensus recommends that for average-risk individuals, screening should start after the age of 45 or 50 years, varying between different governing bodies and scientific societies [64,65,66,67,68,69,70]. Annual or biennial guaiac or fecal occult blood testing (FOBT), colonoscopy every 10 years, or flexible sigmoidoscopy every 5 years are the most recommended modalities, with CTC offering another option. Colonoscopy being the reference standard for colonic polyp detection places this investigation as the examination of choice for CRC screening. In a single procedure, polyps can be removed and tissue biopsied, with the possibility for removing tissue during the same procedure [71,72].

Population screening should be safe, readily available, cause minimal discomfort, free of serious adverse events, repeatable, and affordable. Colonoscopy is not an ideal screening modality. While considered the gold standard for polyp detection, polyp miss rates of 22–28% have been reported [73]. Serious adverse events are rare but include sedation-related complications, bowel perforation, cardiovascular complications, patient anxiety about the discomfort of the examination, and embarrassment [74]. Furthermore, not infrequently, colonoscopy may not be complete. Incomplete colonoscopy is often a result of non-passable segments, stenosis or other obstacles of passage, and/or patient intolerance [75].

Occult blood testing using the FIT is simple to administer and is currently implemented as the primary screening modality for early detection of advanced colorectal neoplasia [76]. A positive test triggers a recommendation for conventional colonoscopy. False positive FIT tests relate to stool concentration, with sensitivity increasing and specificity decreasing with higher cut-off levels [77]. The test cannot distinguish bleeding arising from advanced neoplasia or from non-neoplastic disorders, including peptic ulceration, inflammatory bowel, and hematological diseases [78]. The FIT cut-off value should aim to optimize sensitivity and specificity. There is evidence that at cut-off values of 45, 80, 125, 175, and 350 ng Hb/mL the number of colonoscopies required to detect one advanced neoplasm would be 24, 19, 16, 14, and 10, respectively. Given that conventional colonoscopy is capital- and labor-intensive, invasive, and associated with rare serious adverse events, offering diagnostic colonoscopy to patients with a positive FIT test is highly inefficient, with large numbers of negative colonoscopies draining resources and exposing patients with negative studies to the procedure and potential complications. In attempts to determine optimal cut-off points for triggering a colonoscopy, proposals have ranged from 45–150 ng Hb/mL. However, there remain a small subgroup of patients with levels of 10–45 ng/mL where advanced neoplasia will be missed, and for this reason, NHS England has proposed a clinical evaluation, offering CCE to individuals with FIT concentrations in the 10–99 ng Hb/mL range [76,77,78].

### 5.1. Colon Capsule Endoscopy in Patients with Positive FIT

In a 2014 publication, Holleran et al. calculated that for every million participants in the UK FIT screening program, referral for colonoscopy would result in 6000 negative studies. They suggested the model of a “filter test”, which would act as an intermediary between a positive stool test and colonoscopy. The filter would need similar detection rates as colonoscopy but would be minimally invasive and readily accessible. As CCE-2 fulfills these parameters, a prospective study was undertaken to assess the value of CCE-2 followed within 24 h by conventional colonoscopy in individuals returning a positive FIT ≥ 100 ng Hb/mL. CCE detected polyps (any type) in 69% of patients. Overall, the sensitivity, specificity, PPV, and NPV of CCE for any polyp compared with optical colonoscopy was 95%, 65%, 79%, and 90%, respectively [26]. The authors concluded that using CCE filtering in this enriched population could reduce the number needed to colonoscope by 71%.

In 2020, Pecere et al. examined the diagnostic accuracy of CCE-2 for the detection of advanced neoplasia in 178 FIT positive individuals, all of whom underwent traditional colonoscopy within 24 h of completing the capsule study [45]. Advanced neoplasia was defined as an adenoma >10 mm, villous component >20%, high-grade dysplasia, and/or invasive cancer. When using the 6-mm cutoff, CCE-2 sensitivity and specificity for advanced neoplasia was 90% and 66.1%, respectively, with a 57.4% positive predictive value (PPV) and 92.9% negative predictive value (NPV). At a 10-mm cut-off, the equivalent calculations were 76.7%, 90.7%, 80.7%, and 88.4%, respectively. CCE-2 detected 8 of 9 colorectal cancers, and of 41 false positives at the 6-mm cut-off, 32 (89%) had non-advanced cancers upon colonoscopic examination. The authors concluded that in this enriched population, CCE-2 has a high sensitivity for detecting advanced adenomas at the 6-mm cut-off and a low specificity resulting from the definition of advanced neoplasia [45].

In 2021, Vuik et al. reported a systematic review of CCE, comprising 13 studies and 2485 patients. The polyp detection rate for CCE-2 was 24–74%. For polyps ≥ 6 mm, the sensitivity of CCE was 79–96% and the specificity was 66–97%. For polyps ≥ 10 mm, the sensitivity of CCE was 84–97%. Notably, the CRC detection rate for completed CCEs was 93% [57]. These studies, which were primarily designed to explore the use of CCE as a filter to colonoscopy following a positive FIT test, indicate that CCE appears to be an effective modality for the detection of CRC and polyps with accuracy comparable to colonoscopy and superior to CTC. From the systematic review, the authors provided further evidence that CCE can contribute to the scaling of CRC screening programs.

In the interim analysis of the Danish CareForColon2015 trial, the initial 234 CCEs were evaluated for quality, safety, and completion rate. The completion rate for CCEs was 67.9% and the rate of conclusive investigations was 80.3%. The polyp detection rate was 73.5%, and six suspected cancers were identified (2.6%). The authors discussed the satisfactory outcomes, but concluded that the lower-than-expected proportion of suspected cancers would be followed-up, and explored ways to improve the completion rate [50].

### 5.2. Participation Rate and Uptake of Colon Capsule Endoscopy

Participation rate is pivotal in population-based screening programs. The desirable threshold set in the European guidance is >65%. Offering FIT-based screening in 21 European countries, the overall participation was 49.5% [79,80]. This raises the question whether offering CCE as a screening tool might contribute to an increased participation rate. In 2019, Thygesen et al. reported a mixed methods study of positive FIT individuals that offered both home-based CCE and inpatient OC. Participants were then asked to respond to a questionnaire designed to assess discomfort during CCE and colonoscopy. In addition, 10 of the 253 included patients were invited to engage in a semi-structured interview about their experience. The patients reported that the less invasive CCE examination caused less pain and embarrassment, but the disadvantages included the large capsule that had to be swallowed, the longer wait for the results, and the need for conventional colonoscopy if significant pathology was reported [74]. The uptake of CCE vs. optical colonoscopy within first-degree relatives of patients with CRC was also studied by Adrian de Ganzo et al. It was demonstrated that the uptake was similar between the CCE and colonoscopy (55.8% vs. 52.2%), but the crossover rate was higher from the CCE group (57.4%) than from the colonoscopy group (30.2%). The latter may be attributed to patients’ unwillingness to undergo bowel preparation twice if pathology was found at CCE, which would require subsequent colonoscopy [81]. In a recent meta-analysis by Deding et al., the authors assessed patient-reported outcomes and preferences for CCE and conventional colonoscopy. Although pooled patient preferences were estimated to be 52% (CI 95%: 41–63%) for CCE and 45% (CI 95%: 33–57%) for conventional colonoscopy, not indicating a significant difference, the tolerability for CCE was consistently reported to be higher or equal to that of conventional colonoscopy [58].

## 6. Limitations in the Use of Colon Capsule Endoscopy

### 6.1. Colonic Visualization (Bowel Preparation) and Completion Rate

When evaluating CCE as a diagnostic modality, the completion rate and quality of bowel preparation require consideration. In their 2016 meta-analysis, Spada et al. reported an 81% rate of adequate cleansing for CCE-2 and a 90.5% completion rate [53]. In their 2020 meta-analysis of 26 relevant studies, Deding et al. reported a CCE completion rate ranging from 65–93%, with a pooled estimate of 0.76 (95% CI, 68–84%) [82]. Currently, the recommended bowel preparation is the same as for conventional colonoscopy [83,84,85]. Vuik et al., in the large systematic review of 13 studies and nearly 2500 patients, reported a completion rate of nearly 90% when a sodium phosphate booster was used [57].

Conversely, in a prospective French study involving 689 CCEs, the completion rate and adequate bowel preparation was less promising. In particular, less than 50% of the examinations were both complete and with adequate bowel preparation; and the authors concluded that improvement would be needed to increase the reliability of CCE, since most of the missed colonic advanced neoplasia were due to incomplete CCE with distal neoplasia location [86]. In analogy to this, the previously discussed Danish trial also reported a lower-than-expected completion rate, and discussed ways to optimize that [50]. In the 2021 meta-analysis by Kjølhede et al., the rate of patients with adequate bowel preparation varied from 40% to 100%, and the rates of complete CCE transit varied from 57% to 100%. The researchers argued that the relatively high rate of incomplete investigations limits the application of CCE in a CRC screening setting, despite the good polyp detection rate [54].

The Danish group investigated whether the prokinetic prucalopride, increases the completion rate of CCE. They found that the completion rate was 74.9% in the prucalopride group and 56.7% in the control group (standard preparation group). Additionally, the mean CCE transit time was 2 h and 8 min faster in the prucalopride group. This did not appear to negatively affect the detection rate of CCE: 589 polyps (mean 2.9) were found in the prucalopride group compared to 522 polyps (mean 2.6) in the control group [87].

### 6.2. A Cased of Missed Colorectal Cancer

Although most cases of missed colon lesions were linked to inadequate bowel preparation or incomplete examinations, MacLeod et al. recently described an interesting case of a patient that underwent CCE. The CCE reported 17 polyps. Notably, a 40-mm cecal pole tumor, which was not detected by the CCE, was identified at follow-up colonoscopy, and this was surgically resected, thereby confirming a moderately-differentiated adenocarcinoma. The tumor was not definitively identified on retrospective review of the CCE images either [88].

### 6.3. Observer Error Reporting CCE

Few colon capsule practitioners have fulfilled the recommendations for formal capsule endoscopy training [89]. Buijs et al. has examined the intra- and inter-observer agreement in evaluating CCE videos. Three experienced and two beginner observers reviewed a total of 42 complete CCE videos. There was moderate agreement among experts on the detection of large polyps and the number of polyps identified. Beginners were in moderate agreement with experts on polyp detection. Intra-observer agreement among experts was moderate to excellent for the detection of large polyps (≥10 mm) and excellent for the number of polyps. Intra-observer agreement in beginners was poor to moderate for all variables [90]. These findings emphasize the need for CCE reader training based on formative mentoring and a summative assessment.

### 6.4. Other Limitations in Colon Capsule Endoscopy Use

While CCE is safe, has high patient acceptability, and ever improving sensitivity and specificity for polyp detection, there are further limitations that need consideration. It has already been discussed that inadequate bowel preparation and/or completion rate still pose a challenge and would require further optimization. Additionally, most of the studies conducted to date involved non-Asian participants, and this should be acknowledged. In analogy to this, CCE’s usage range was different in different areas, such as having less usage in Asia.

Additionally, the relatively long reading time of CCE should also be taken into consideration. The reading speed is dictated by the overall circumstances. The realistic overall average reading time is between 30 and 60 minutes, and a second opinion may be wise and welcome for difficult frames [91]. It is likely, however, that computer-aided diagnosis will complete a pre-read and substantially reduce reporting time [92,93]. Increasing CCE delivery requires training large numbers of accredited readers. Several residential training programs exist for small bowel capsule endoscopy, but there are few CCE mentoring opportunities for hands-on training that is required to achieve capsule endoscopy competency [89].

### 6.5. Cost-Effectiveness of Colon Capsule Endoscopy

The procedure remains a relatively expensive examination, especially in the absence of shifting manpower resources from traditional endoscopy to frontline CCE examination. An older study attempted to provide a model to assess the cost-effectiveness of population-based screening for CRC using CCE and to compare the cost-effectiveness with that of a colonoscopy screening program. The incremental cost-effectiveness (compared with no screening) of colonoscopy and capsule endoscopy was $16,165 USD and $29,244 USD per life-year saved, respectively. Nevertheless, one should consider that these data likely cannot be extrapolated to the current CCE-2 era. Additionally, the authors discussed that simulating an initial compliance to capsule endoscopy was 30% better than colonoscopy, thus capsule endoscopy became the more effective and more cost-effective option [94]. Further up-to-date studies would be required to further investigate and validate such results.

## 7. Adverse Events Linked to Colon Capsule Endoscopy

Regarding clinically-significant adverse events, in their 2016 meta-analysis, Spada et al. demonstrated that CCE is extremely safe, with no serious adverse events occurring in over 2000 subjects. They reported a cumulative adverse-event rate of 10.4%, which was mostly linked to bowel preparation (cumulative rate 9.7%), while those directly associated with CCE yielded a cumulative rate of 0.33% [53]. In their systematic review, Vuik et al. reported that no CCE-related adverse events occurred in any of the studies included in the meta-analysis [57]. Likewise, in a retrospective study, Fernández-Urién et al. reported an overall incidence of colon capsule-related adverse events of less than 0.5% [95]. A recent study assessing the symptomatic patient perspective showed that the main cause of patient dissatisfaction with CCE was bowel preparation. Interestingly, 77.5% of patients would prefer a CCE if they required further bowel investigation (over a colonoscopy) [96]. In a large meta-analysis assessing the adverse events of video capsule endoscopy (including CCE), the adverse-event rate was shown to be very low [97]. Lastly, Cortegoso Valdivia et al. also conducted a meta-analysis to study the indications, detection, completion, and retention rates of capsule endoscopy over the last two decades. Their study involved the assessment of all the different types of capsule. Among all different capsule types, for pooled indications, the lowest retention rate was found for CCE (RR = 0.008) (and magnetically-controlled capsule endoscopy) [98]. These studies, alongside a series of others, emphatically illustrate that CCE is a safe and well-tolerated procedure.

## 8. Colon Capsule Endoscopy vs. CT Colonography

There have been a series of studies that have resulted in recognition that CT colonography (CTC) is an acceptable modality for colon polyp diagnosis, especially in the setting of incomplete colonoscopy [68,69,70,85,99,100]. There have also been several reports comparing CTC with CCE [27,30,41].

The VICOCA study reported a randomized trial of 290 FIT positive individuals that were referred for colonoscopy who agreed to undergo either CCE or CTC prior to colonoscopy. The sensitivity, specificity, and positive and negative predictive values for clinically-significant polyps were 98.1%, 76.6%, 93.7%, and 92.0% in the CCE group and 64.9%, 95.7%, 96.8%, and 57.7% in the CTC group. The detection rate for advanced colorectal neoplasm was higher in the CCE group than in the CTC group (100% vs. 93.1%; *p* = 0.08). Both CCE and CTC identified all patients with cancer. CCE detected more patients with any lesion than CTC (98.6% vs. 81.0%, respectively; *p* = 0.002). The authors concluded that both techniques are similar in detecting advanced colorectal neoplasia, but CCE is more sensitive for the detection of any neoplastic lesion [43].

In the recently published TOPAZ study, patients were randomized to CCE or CTC and subsequent blinded colonoscopy. The sensitivity and specificity of CCE for polyps ≥6 mm was 79.2% and 96.3%, while that of CTC was 26.8% and 98.9%. The sensitivity and specificity of CCE for polyps ≥10 mm was 85.7% and 98.2% compared with 50% and 99.1% for CTC. The authors concluded that CCE was superior to CTC for the detection of polyps ≥6 mm and non-inferior for those ≥10 mm, and suggested that CCE should be considered comparable or superior to CTC as a colorectal neoplasia screening test—but argued whether any of these tests are as effective as conventional colonoscopy [41].

In their recent meta-analysis, Deding et al. reported the efficacy of CCE (both 1st- and 2nd-generation procedures were included) vs. CTC following incomplete colonoscopy. The polyp yields of CTC and CCE were 10% and 37% for polyps of any size, 13% and 21% for polyps ≥5 mm, and 4% and 9% for polyps ≥10 mm polyps. Overall, the diagnostic yield of CCE for polyps of any size was almost fourfold compared to CTC [82].

In summary, published studies concur that compared to CTC, second-generation CCE has comparable or even superior accuracy in detecting colonic polyps, and that both are preferred by patients to conventional colonoscopy. Both CCE and CTC are almost free of serious procedure-related adverse events, although radiation exposure may be a long-term consideration. 

## 9. International Guidance on the Role of Colon Capsule Endoscopy

CCE-2 has been endorsed by some major scientific societies and governing bodies. The US Food and Drug Administration has approved CCE-2 as an adjunctive test in patients following incomplete colonoscopy and in the diagnostic evaluation of patients with suspected lower gastrointestinal bleeding [70]. Recently, the European Society for Gastrointestinal Endoscopy and the European Society of Gastrointestinal and Abdominal Radiology updated their recommendations to integrate all advances in the field. While CCE is not recommended as a first-line screening investigation, they recommended that CCE-2 may be considered when colonoscopy is incomplete or contraindicated [85]. In Japan, CCE is recommended for incomplete colonoscopy [101]. Based on the large body of evidence indicating high sensitivity for CCE, in March 2021, NHS England approved the use of colon capsule endoscopy as a first-line investigation of low-risk patients with a stool FIT test between 10–100 ng Hb/mL [102].

There is consensus that CCE contraindications are the same as those for small bowel capsule endoscopy. The examination should not be performed in patients with swallowing difficulty, those likely to be intolerant of the bowel cleansing and booster preparations, and individuals with known or suspected gastrointestinal obstruction, stricture, or fistula. Relative contraindications include patients fitted with cardiac pacemakers, or other implanted electromedical devices, and pregnant women [101].

## 10. Discussion

A number of carefully controlled studies concur that CCE-2 has excellent sensitivity for polyp detection and a high negative predictive value. Like CTC, capsule screening presents the clinician with a decision on the threshold for colonoscopy referral. Pickardt et al. reported a carefully constructed CTC decision analysis that is readily extrapolated to CCE [103]. The model assumed that CRC risk was independent of advanced adenoma size. The analysis derived the number of diminutive (≤5 mm), small (6–9 mm), and large (> or 10 mm) polyps requiring removal to detect one advanced adenoma or prevent one CRC over a 10-year period. Several assumptions were made. The ten-year cancer risk was modelled in relation to polyp size and age of diagnosis. Their model assumed that CRC prevention was related to the discovery of advanced adenomas, defined by a size of ≥10 mm, or histological analysis indicating a prominent villous component or high-grade dysplasia [104]. The prevalence data for polyps according to both size and histology was derived from a cohort of asymptomatic adults who underwent both CTC and same-day colonoscopy as part of a published screening trial [100,105,106]. The model assumed an initial CTC screening age of 60 years and a polyp prevalence of 4.5% [107]. Based on the National Polyp Study [108], the model assumed that 76% of CRC is preventable by detection and removal of advanced neoplasms, with a 24% residual risk due to missed lesions or derived via a pathway distinct from the adenoma–carcinoma sequence [109]. The model estimated a 10-year CRC risk for unresected diminutive, small, and large polyps as 0.08%, 0.7%, and 15.7%, respectively, and the number of diminutive, small, and large polyps needing removal to avoid one advanced adenoma was 562.0, 71.0, and 2.5, respectively, with 2,352,297.0 and 10.7 polypectomies required to prevent one CRC over 10 years. The authors concluded that over a 10-year period, there is a very low or low likelihood of advanced neoplasia in patients presenting with diminutive or small polyps, respectively, while referral of large CTC-detected polyps for polypectomy is highly effective.

In a literature review of diminutive and small colon polyp management, Coe and Wallace concluded that both retrospective and prospective studies indicate that in colorectal polyps < 10 mm in size, there is an exceptionally low prevalence of advanced pathology [110]. In addition, natural history studies suggest these polyps exhibit little or slow growth, and in some cases, regress. Like Pickardt, they concluded that by balancing the risks of colonoscopy and polypectomy with cancer, the discovery of diminutive or small polyps might follow a non-interventional pathway without reducing cancer prevention effectiveness. Applying similar reasoning to the discovery of diminutive or small polyps on CCE, it is reasonable to suggest that colon capsule endoscopy could be included as a first-line choice for polyp screening.

## 11. Conclusions

Population-based stool occult blood testing followed—if positive—by colonoscopy has made a considerable impact on colon cancer mortality. The test fulfills the criteria for population screening; primary screening using colonoscopy offers diagnostic accuracy and the advantage of immediate polyp removal. However, primary colonoscopy screening fails to meet the acceptable criteria for population screening. The procedure, in asymptomatic individuals, requires day-case admission, causes discomfort sufficient to require analgesia and sedation, and is rarely associated with devastating complications. While considered the gold standard for detecting colorectal neoplasia, this is balanced by large numbers of negative procedures required to diagnose a single advanced neoplasm.

CCE is a new device in gastroenterology with potential to change the colorectal screening landscape. Ambulant individuals swallow the capsule with a sip of water, the procedure is pain free, and, after taking the capsule, individuals can return home unaccompanied, where the capsule can complete its mouth-to-toilet journey. CCE is currently well placed to straddle the current models of occult blood testing and colonoscopy. This minimally invasive procedure asks and answers the question, “who needs a colonoscopy?”, and offers patients a safe, effective, and gentler screening option.

## Figures and Tables

**Table 1:** Technical characteristics of 2nd-generation colon capsule.

PillCam™ COLON 2 Capsule Specifications *		
Physical	Size (mm)	32.3 × 11.6
	Weight (g)	2.9
Optical	Number of heads	2
	Illumination	4 white light emitting diodes on each side
	Viewing direction	longitudinal
	Field of view (°)	172
Operational	Operation time	Minimum of 10 h
	Frame rate (f/sec)	Up to 35

* Available online: https://www.medtronic.com/covidien/en-us/products/capsule-endoscopy/pillcam-colon-2-system.html (accessed on 27 June 2022).

**Table 2:** Overview of recent systematic reviews and meta-analyses on colon capsule endoscopy and colon polyp detection.

Study	Year of Publication	Number of Studies Included (Number of Patients)	Polyp Detection Rate (≥6 mm)	Polyp Detection Rate (≥10 mm)	Completion Rate (%)	Adequate Bowel Preparation (%)	Adverse Events (%)	Adverse Events (Type)
**Sulbaran et al.** **[41]**	2022	8 (1602)	*sensitivity:* 88% (95% CI: 84–91%) *specificity:* 94% (95% CI: 92–95%)	*sensitivity:* 88% (95% CI: 82–93) *specificity:* 95.5% (95% CI: 94–97%)	88.42	54–92	7.8	Mild–mostly related to bowel preparation
**Ali et al.** **[42]**	2021	5 (1305)	*sensitivity:* 86% (95% CI: 82–91%) *specificity:* 88% (95% CI: 72–96%)	*sensitivity:* 86% (95% CI: 80–91%) *specificity:* 96% (95% CI: 92–98%)	91.6	82	-	-
**Spada et al.** **[53]**	2016	14 (2420) *	*sensitivity*: 86% (95% CI: 82–89%) *specificity:* 88.1% (95% CI: 74.2–95%)	*sensitivity*: 87% (95% CI: 81–91%) *specificity:* 95.3% (95% CI: 91.5–97.5%)	90.5	81	10.4	Most related to bowel preparation—in 0.33% mild to moderate related to colon capsule
**Kjølhede et al.** **[54]**	2021	12 (1898)	*sensitivity:* 87% (95% CI: 83–90%) *specificity:* 88% (95% CI: 75–95%)	*sensitivity:* 87% (95% CI: 82–90%) *specificity:* 95% (95% CI: 92–97%)	57–100	40–100	Up to 25%	Mild (such as nausea and vomiting) related to bowel preparation–few cases of capsule retained in the caecum
**Möllers et al.** **[55]**	2021	13 (2328) †	Sensitivity: 87;% (95% CI: 83–90%) Specificity: 87% (95% CI: 76–93%)	Sensitivity: 87% (95% CI: 83–90%) Specificity: 95% (95% CI: 92–97%)	61–92	-	10.3	Mostly mild, and mainly related to the bowel preparation
**Vuik et al.** **[57]**	2021	13 (2485)	Sensitivity: 79–96% Specificity: 66–97%	Sensitivity: 77–97% Specificity: 91–99%	57–92	70–92	-	No colon capsule endoscopy-related adverse events–use of bowel preparation not associated with severe adverse events
**Deding et al.** **[58]**	2020	26 (2620) §	Diagnostic yield: 8–41%; Pooled estimate: 21% (95% CI 12–32%).	Diagnostic yield: 3–22%, Pooled estimate: 9% (95% CI 3–17%).	76	90	-	-

* Meta-analysis included colon capsule endoscopy-1 (*n* = 1128) and colon capsule endoscopy-2 (*n* = 1292); results on polyp detection rate, completion rate, and quality of bowel preparation depicted in this table are only relevant to colon capsule endoscopy-2. † 13 studies included in the systematic review and 9 in the meta-analysis. § This is the total number of included studies, some of them assessing CT colonography only. The number of studies and participants relevant to colon capsule endoscopy are 8 and 553, respectively.
